# Development of prediction models to estimate extubation time and midterm recovery time of ophthalmic patients undergoing general anesthesia: a cross-sectional study

**DOI:** 10.1186/s12871-023-02021-3

**Published:** 2023-03-17

**Authors:** Xuan Huang, Ronghui Tan, Jian-Wei Lin, Gonghui Li, Jianying Xie

**Affiliations:** 1 Joint Shantou International Eye Centre of Shantou University and the Chinese University of Hong Kong, Shantou, Guangdong China; 2 Shantou University Medical College, Shantou, Guangdong China

**Keywords:** Delayed Emergence from Anesthesia, Prediction Model, Fuzzy Neural Network, Extubation Time, Midterm Recovery Time, Risk Factors

## Abstract

**Background:**

To develop prediction models for extubation time and midterm recovery time estimation in ophthalmic patients who underwent general anesthesia.

**Methods:**

Totally 1824 ophthalmic patients who received general anesthesia at Joint Shantou International Eye Center were included. They were divided into a training dataset of 1276 samples, a validation dataset of 274 samples and a check dataset of 274 samples. Up to 85 to 87 related factors were collected for extubation time and midterm recovery time analysis, respectively, including patient factors, anesthetic factors, surgery factors and laboratory examination results. First, multiple linear regression was used for predictor selection. Second, different methods were used to develop predictive models for extubation time and midterm recovery time respectively. Finally, the models’ generalization abilities were evaluated using a same check dataset with MSE, RMSE, MAE, MAPE, R-Squared and CCC.

**Results:**

The fuzzy neural network achieved the highest R-Squared of 0.956 for extubation time prediction and 0.885 for midterm recovery time, and the RMSE value was 6.637 and 9.285, respectively.

**Conclusion:**

The fuzzy neural network developed in this study had good generalization performance in predicting both extubation time and midterm recovery time of ophthalmic patients undergoing general anesthesia.

**Trial registration:**

This study is prospectively registered in the Chinese Clinical Trial Registry, registration number: CHiCRT2000036416, registration date: August 23, 2020.

**Supplementary Information:**

The online version contains supplementary material available at 10.1186/s12871-023-02021-3.

## Background

Delayed emergence from anesthesia (DEA) is described as an abnormally slow pace state of regaining consciousness after general anesthesia, often characterized by persistent somnolence. In clinical practice, delayed emergence is defined as the failure to regain consciousness 30 ~ 60 min after general anesthesia [1, 2]. DEA is a common complication after general anesthesia, and also one of the main causes for endangering patient safety [3]. studies had showed the incidence rate of DEA was between 8.1% to 50.5% [4, 5]. Anesthetic drugs usages, improper management or practice during anesthesia, surgical factors [6] and patient’s individual variation [7, 8] are risk factors.

The recovery of general anesthesia patients can be generally divided into three stages. Firstly, the early recovery stage, generally refers to the period from the end of anesthesia to the regain of consciousness, airway protective reflex and motor function. At this stage, patient is prepared for endotracheal tube removal. Secondly, the midterm recovery stage, generally refers to in-patients who meet the post-anesthetic care unit (PACU) discharge standards and are admitted to the general ward or out-patients who meet the standards can be discharge home. Finally, the late-term recovery stage is when the patient achieves full physical and mental recovery and is able to resume daily activities [9, 10].

Due to the large amount of data generated during perioperative surgery and anesthesia management, anesthesiology is very suitable for cross-research and application of artificial intelligence (AI), such as machine learning deep learning and data mining etc.[11]. A number of studies had revealed that AI has shown excellent performance in preoperative evaluation, risk prediction, decision system and other studies in the field of anesthesia, and has great development potential [12–14]. However, few study has focused on the problem of delayed awakening from anesthesia. Xie et al. [15] had developed a model using clinical and pharmacogenetics data to predict recovery time from general anesthesia but it required extra venous blood sample for genetic analysis, which was an additional medical burden for both patients and staffs, and it may not be practical for hospital with large surgical amount. Therefore, in this study, we try to develop model using perioperative data of electronic medical record system to predict general anesthesia ophthalmic patient’s extubation time and midterm recovery time, so as to help staffs identify critical timing and carry out timely interventions, which can eventually shorten patients’ early and midterm recovery time, reduce postoperative complications and optimize the PACU utility.

## Methods

### Data source

Retrospectively collected the perioperative information of patients underwent general anesthesia and ophthalmic surgery from January 2018 to October 2020 in Joint Shantou International Eye Center (JSIEC) Shantou University & the Chinese University of Hong Kong in accordance with the Declaration of Helsinki principles. This study was approved by the Shantou International Eye Centre of Shantou University and The Chinese University of Hong Kong Academic Board and Medical Ethics Board, approval number: EC20200728(8)-P06. Because the retrospective nature of the study, informed consent was waived.

The inclusion criteria were set as: patient age from 4 ~ 65 years old; using anesthetic technique of total intravenous anesthesia; complete patient perioperative information. The exclusion criteria were set as: using anesthetic techniques except total intravenous anesthesia; American Society of Anesthesiologists Physical Status Classification System [16] (ASA grade) > 2; patient with history of psychotropic drug use and mental illness; patient with postoperative continuous sedation or transferred into intensive care unit.

### Outcomes

#### Extubation time

We defined extubation time as time from discontinuation of anesthetic medication to immediate removal of endotracheal intubation/laryngeal mask. Extubation timing was evaluated comprehensively by registered anesthesiologist and PACU nurses according to patient’s spontaneous ventilation, consciousness regaining level, body movement and other extubation indications [17, 18], and a registered anesthesiologist decided whether to remove patient’s endotracheal intubation/laryngeal mask or not.

#### Midterm recovery time

We defined midterm recovery time as time from discontinuation of anesthetic medication to patient’s Steward score [10, 19] reached 6 points and met the criteria for transfer out of the PACU or operating room.

### Risk factors collection

After literature reviews and clinical experience summarization, factors that may affect post-anesthesia awakening and midterm recovery were categorized in Table 1. All the laboratory examinations were performed by the hospital clinical laboratory.

**Table 1:** Related factors used for extubation and midterm recovery time predictor analysis

Type variables	Variable name
patient factors (8)	Gender (male/female), Age, Weight (kg), Height (cm), Body mass index (kg/m^2^),Preoperative body temperature (℃), Underlying diseases (yes/no), Postoperative body temperature (℃)
anesthetic factors (18)	Preoperative atropine (mg), ASA grade (degree1/degree2), Transfusion volume (ml), Fentanyl (μg), Total amount of propofol(mg), Muscle relaxant types (atracurium/cisatracurium), Total amount of remifentanil (μg), Dexmedetomidine usage (yes/no), Ondansetron usage (yes/no), Nalbuphine (mg), Dexamethasone (mg),Tidal volume (ml), End-tidal CO2 (mmHg), Anesthesiologists, Intraoperative atropine (mg), Anesthesia time (min), Extubation time* (min), Postoperative complications** (yes/no)
surgery factors (5)	Timing of surgery (selective/emergency), operation history (yes/no), surgery time (min), surgeons, surgery types
laboratory examination results (56)	Serum potassium (mmol/L), Serum sodium (mmol/L), Serum chlorine (mmol/L), Serum calcium (mmol/L), Serum phosphorus (mmol/L), Total carbon dioxide (mmol/L), Total protein (g/L), Albumin (g/L), Globulin (g/L), ALB, Total bilirubin (μmol/L), Direct bilirubin (μmol/L), Indirect bilirubin (μmol/L), Alanine aminotransferase (U/L), Aspartate aminotransferase (U/L), Glutamyl transpeptidase (U/L), Alkaline phosphatase (U/L), Cholinesterase (U/L), L-lactate dehydrogenase (U/L), α -hydroxybutyrate dehydrogenase (U/L), Creatine kinase (U/L), Creatine kinase isoenzyme MB (U/L), Ureophil (mmol/L), Creatinine (μ mol/L), Uric acid (μ mol/L), Cystine protease inhibitor C (mg/L), Glucose (mmol/L), Fructosamine (mmol/L), Total cholesterol (mmol/L), Triglyceride (mmol/L), High density cholesterol (mmol/L), Low density cholesterol (mmol/L), C-reactive protein (mg/dL), White blood cell count (10^9/L), Neutrophil percentage (%), Lymphocyte percentage (%), Monocyte percentage (%), Eosinophil percentage (%), Basophils percentage (%), Neutrophil absolute value (10^9/L), Lymphocyte absolute value (10^9/L), Monocyte absolute value (10^9/L), Eosinophil absolute value (10^9/L), Basophil absolute value (10^9/L), Red blood cell count (10^12/L), Hemoglobin (g/L), HCT (%), Mean corpuscular volume (fL), Mean corpuscular hemoglobin (pg), Mean corpuscular hemoglobin concentration(g/L), Red blood cell distribution width (%), Platelet count (10^9/L), Platelet ratio (%), Mean platelet volume (fL), Platelet distribution width (%), Urine routine (normal/abnormal)

In this study, postoperative complications* are defined as unexpected/unplanned complications related to anesthesia and/or surgery that occurred before patient transferred out the PACU/operating room

Extubation time* and postoperative complications** were only used for midterm recovery time predictors screening

### Sample size

By reviewing literature and consulting with clinical anesthesiologists, 85 factors for extubation time analysis and 87 factors for midterm recovery time analysis were collected. Based on the Kendall criterion [20]: for outcome of extubation time, the sample size should be 1275 ~ 1700, and for outcome of midterm recovery time, the sample size should be 1305 ~ 1740. In this study, a total of 1824 samples were collected, and all sample information were complete without missing values.

### Statistical analysis and models development

#### Predictor analysis

SPSS20.0 (IBM Corp. Released 2013. IBM SPSS Statistics for Windows, Version 22.0. Armonk, NY: IBM Corp.) was used for statistical analysis.

The mean and standard deviation ($\overline{x }$ ± S) was used for statistical description of continuous variables conforming to normal distribution, while the median M (P25, P75) was used for statistical description of continuous variables that does not conforming to normal distribution. Dichotomous variables or ranked data were described by number and composition ratio. Stepwise multivariate linear regression was used to analyze continuous variables and dichotomous variables. Stepwise multivariate linear regression analysis was conducted after dummy variables were set for categorical variables. 0.05 was considered to be statistically significant, and significant predictors were screened out.

#### Model development

##### Dataset division

A randomization seed was set, and dataset was divided into training dataset (*N* = 1276), validation dataset (*N* = 274) and check dataset (*N* = 274) in the ratio of 7:1.5:1.5. The baseline information of the three datasets was compared using variance analysis and chi-square test.

Matlab R2018a (MathWorks®, Natick, Massachusetts, USA) was used to develop fuzzy neural network (FNN), stepwise linear regression model, regression tree model, ensembles of trees regression model, and artificial neural network with different algorithm [Levenberg Marquardt Algorithm (LMA), Bayesian Regularization Algorithm (BRA), Scaled Conjugate Gradient Algorithm (SCGA)] to predict extubation time and midterm recovery time separately.

For FNN, we used Fuzzy Logic Designer to construct Sugeno type fuzzy inference system (FIS), and used Neuro-Fuzzy Designer to develop fuzzy neural network. In the extubation time FNN development, the FIS was using fuzzy C-means algorithm to generate a Sugeno-type FIS. In the midterm recovery time FNN development, the FIS was using subtractive clustering method to generate FIS, the parameter setting was range of influence = 0.45, squash factor = 1.25, accept ratio = 0.78, reject ratio = 0.53.

For regression model (including stepwise linear regression model, regression tree model and ensembles of trees regression model), training dataset and validation dataset are combined into one dataset (*N* = 1550) for regression model training. Regression Learner was used for model development and tenfold cross validation was used for internal validation.

For artificial neural network (LMA, BRA,SCGA), using the same training dataset of regression model, randomly divided into training set, validation set and test set in the ratio of 70%, 15%, 15%, using Kolmogorov theorem [21] for neurons number in the hidden layer calculation: S = 2n + 1 (S: number of neurons in the hidden layer; n: number of neurons in the input layer).

#### Training performance evaluation

Mean square error (MSE), root mean square error (RMSE), goodness of fit (*R*-Squared) and model training time was calculated for training performance evaluation.

#### Generalization performance evaluation

Using the same and separated check dataset (*N* = 274) to perform generalization performance evaluation for each model, MSE, RMSE, mean absolute error (MAE), mean absolute percentage error (MAPE), *R*-Squared and concordance correlation coefficient (CCC) were calculated respectively.

#### Using models in clinical settings

Clinical staffs can use our developed models to predict new patient’s extubation time and midterm recovery time by inputting new patient’s data in Matlab using the calculation code we subjoin in the supplementary file Table S1.

### Flow chart


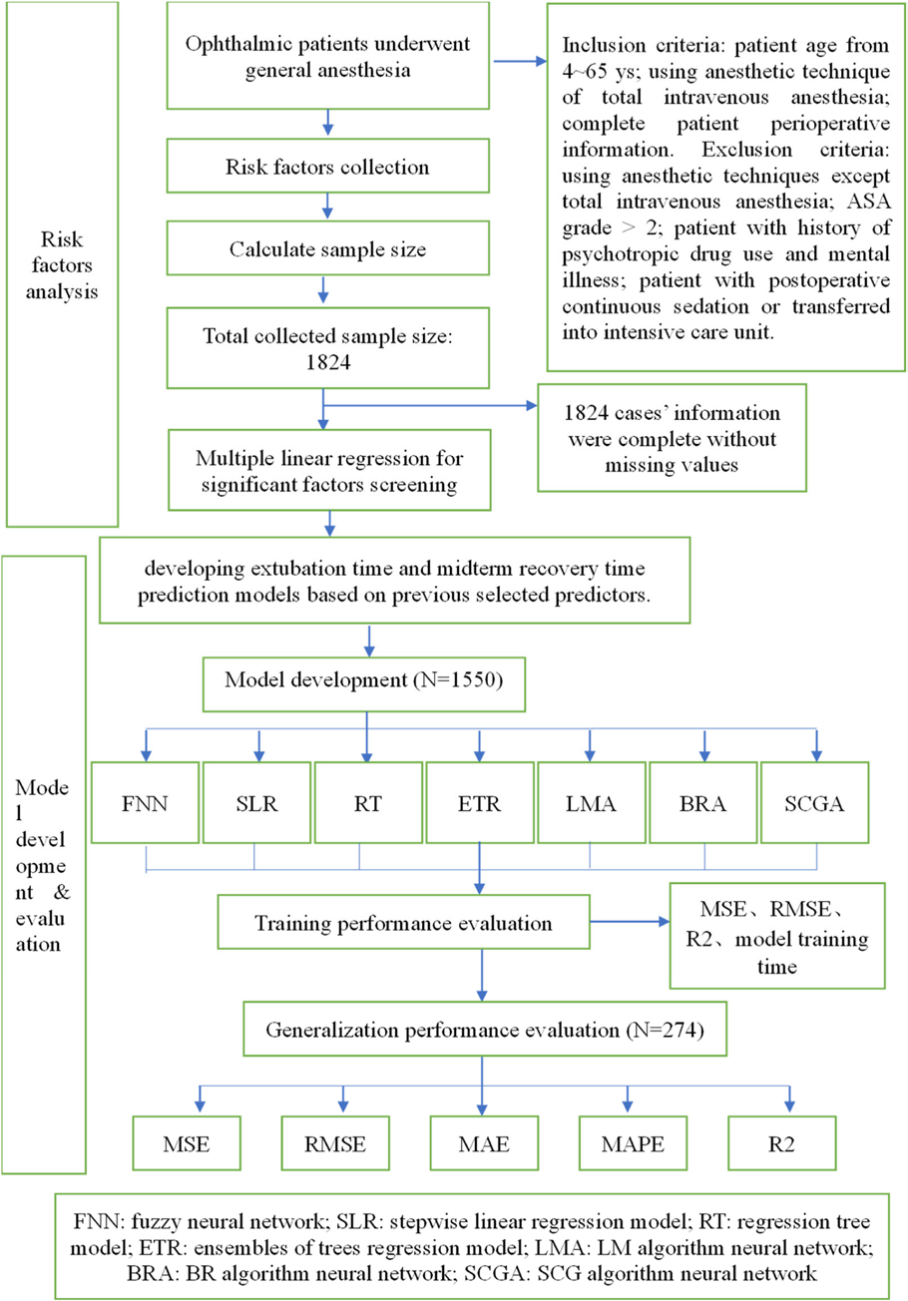


## Results

### Risk factors analysis

A total of 1824 samples were included in this study (for further information, see supplementary file Table S2-S5). The median of extubation time was 55.00 min, with the 25^th^ percentile of 45.00 min and the 75^th^ percentile of 72.00 min. The median of midterm recovery time was 75.00 min, with the 25^th^ percentile of 60.00 min and the 75^th^ percentile of 90.00 min.

By using multiple linear regression analysis to analyze the risk factors of extubation time and midterm recovery time, we obtained 19 risk factors for extubation time and 21 risk factors for midterm recovery time (Table 2). The detail steps of regression analysis are shown in the supplementary file Table S6-S13.

**Table 2:** Predictors of extubation time and midterm recovery time

	Extubation time	Midterm recovery time
Predictors	Anesthesia time	Extubation time
	Creatine kinase isoenzyme MB (CK-MB)	Postoperative body temperature
		Dexamethasone
	Mean corpuscular volume (MCV)	Operation time
	Ondansetron usage	Preoperative atropine
	Mean corpuscular hemoglobin concentration	Nalbuphine
		Preoperative body temperature
	Muscle relaxant types	Transfusion volume
	Preoperative atropine	Red blood cell distribution width
	Nalbuphine	Postoperative complications
	End-tidal CO2 (ETCO2)	Total carbon dioxide
	Cystatin	Underlying diseases
	Urinalysis	Dexmedetomidine usage
	Tidal volume	Ondansetron usage
	Creatinine	ETCO2
	Platelet ratio	Serum total cholesterol
	Transfusion volume	Serum calcium
	Operation history	Muscle relaxant types
	Anesthesiologists	Anesthesiologists
	Surgery types	Surgery types
	Surgeons	Surgeons

### Extubation time prediction model development and evaluation

#### Baseline comparison

The baseline comparison of the training dataset, validation dataset and check dataset for extubation time prediction model is shown in Table 3. Among the 19 predictors included in this study, except for ETCO2 and creatinine, the other 17 predictors had no statistical significance among the training dataset, validation dataset and check dataset (*P* > 0.05).

**Table 3:** Baseline comparison of the training dataset, validation dataset and check dataset for extubation time prediction model

Variable	Training dataset (*n* = 1276)	Validation dataset (*n* = 274)	Check dataset (*n* = 274)	Statistic	Sig.
Anesthesia time (min)	40.00(30.00, 55.00)	45.00 (30.00, 60.00)	40.00 (30.00, 60.00)	2.737	0.065
CK-MB (U/L)	8.00 (4.00, 19.00)	8.50 (3.75, 19.00)	8.00 (4.00, 18.00)	0.421	0.657
MCV (fL)	86.40(82.40, 90.18)	86.40(83.00, 90.43)	86.10(82.00, 89.80)	0.15	0.861
Ondansetron usage (no/yes)	498/778(39.00%/61.00%)	92/182(33.60%/66.40%)	103/171(37.60%/62.40%)	2.866^a^	0.239
Mean corpuscular hemoglobin concentration (g/L)	318.35 ± 19.34	319.94 ± 18.28	320.20 ± 20.70	1.526	0.218
Muscle relaxant types (atracurium/cisatracurium)	789/487(61.80%/38.20%)	180/94(65.70%/34.30%)	159/115(58.00%/42.00%)	3.409^a^	0.182
Preoperative atropine (mg)	0.31(0.21, 0.50)	0.30(0.22, 0.50)	0.40(0.23, 0.50)	0.601	0.549
Nalbuphine (mg)	3.52 ± 1.85	3.62 ± 1.83	3.70 ± 2.07	1.174	0.309
ETCO2 (mmHg)	38.30 ± 2.55	38.07 ± 2.44	37.93 ± 2.36	3.093	0.046
Cystatin (mg/L)	0.79 ± 0.20	0.78 ± 0.20	0.81 ± 0.21	1.561	0.21
Urine routine (normal/abnormal)	930/346(72.90%/27.10%)	198/76(72.30%/27.70%)	206/68(75.20%/24.80%)	0.731^a^	0.694
Tidal volume (ml)	280.00(180.00, 400.00)	265.00(190.00, 400.00)	295.00(190.00, 400.00)	0.883	0.414
Creatinine (μ mol/L)	44.00(36.00, 55.00)	44.00(37.00, 55.00)	45.00(37.00, 59.25)	3.66	0.026
Platelet ratio (%)	0.235(0.208, 0.267)	0.238(0.206, 0.268)	0.233(0.200, 0.265)	0.753	0.471
Transfusion volume (ml)	160.00(120.00, 230.00)	180.00 (130.00, 230.00)	170.00 (130.00, 230.00)	0.826	0.438
Operation history (no/yes)	1132/144(88.70%/11.30%)	244/30(89.10%/10.90%)	241/33(88.00%/12.00%)	0.18^a^	0.914
Anesthesiologists	-	-	-	2.265^a^	0.322
Surgery types	-	-	-	5.527^a^	0.063
Surgeons	-	-	-	1.581^a^	0.454
Extubation time (min)	55.00(45.00, 70.00)	60.00(45.00, 75.00)	60.00(45.00, 75.00)	2.785	0.062

The statistic is the analysis of variance F value, and ^a^stands for the Chi-square test value

#### Training performance evaluation

**Fig. 1:**
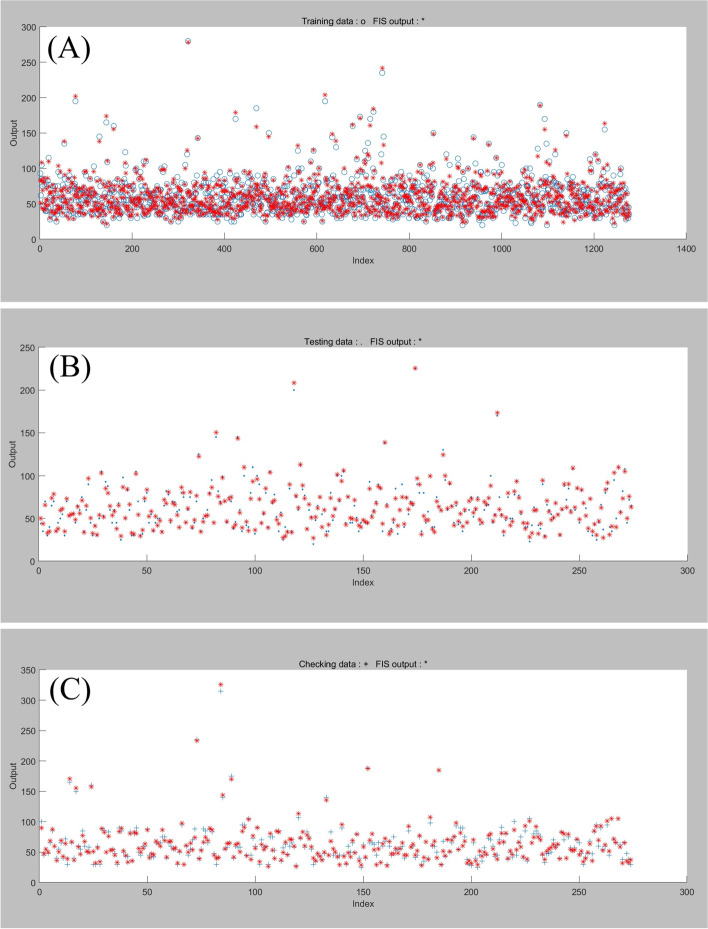
Scatter fitting diagram of predicted and true values of each dataset in extubation time FNN. **A** fitting of the predicted and true value in training dataset; **B** fitting of the predicted and true value in validation dataset; **C** fitting of the predicted and true value in check dataset

**Fig. 2:**
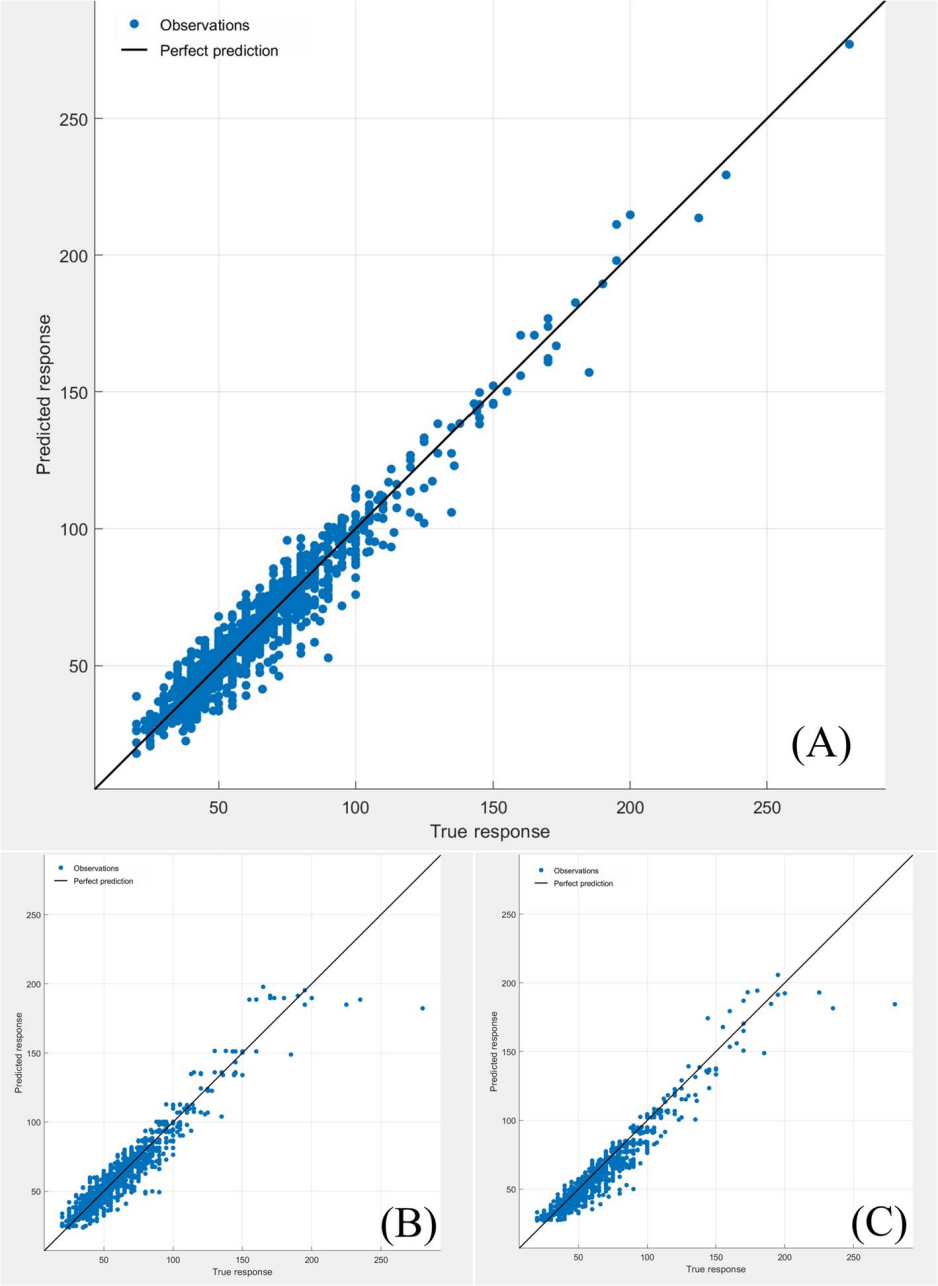
Scatter fitting diagram of predicted and true values in each regression model of extubation time. **A** fitting of the predicted and true value in stepwise linear regression model; **B** fitting of the predicted and true value in regression tree model; **C** fitting of the predicted and true value in ensembles of trees regression model

**Fig. 3:**
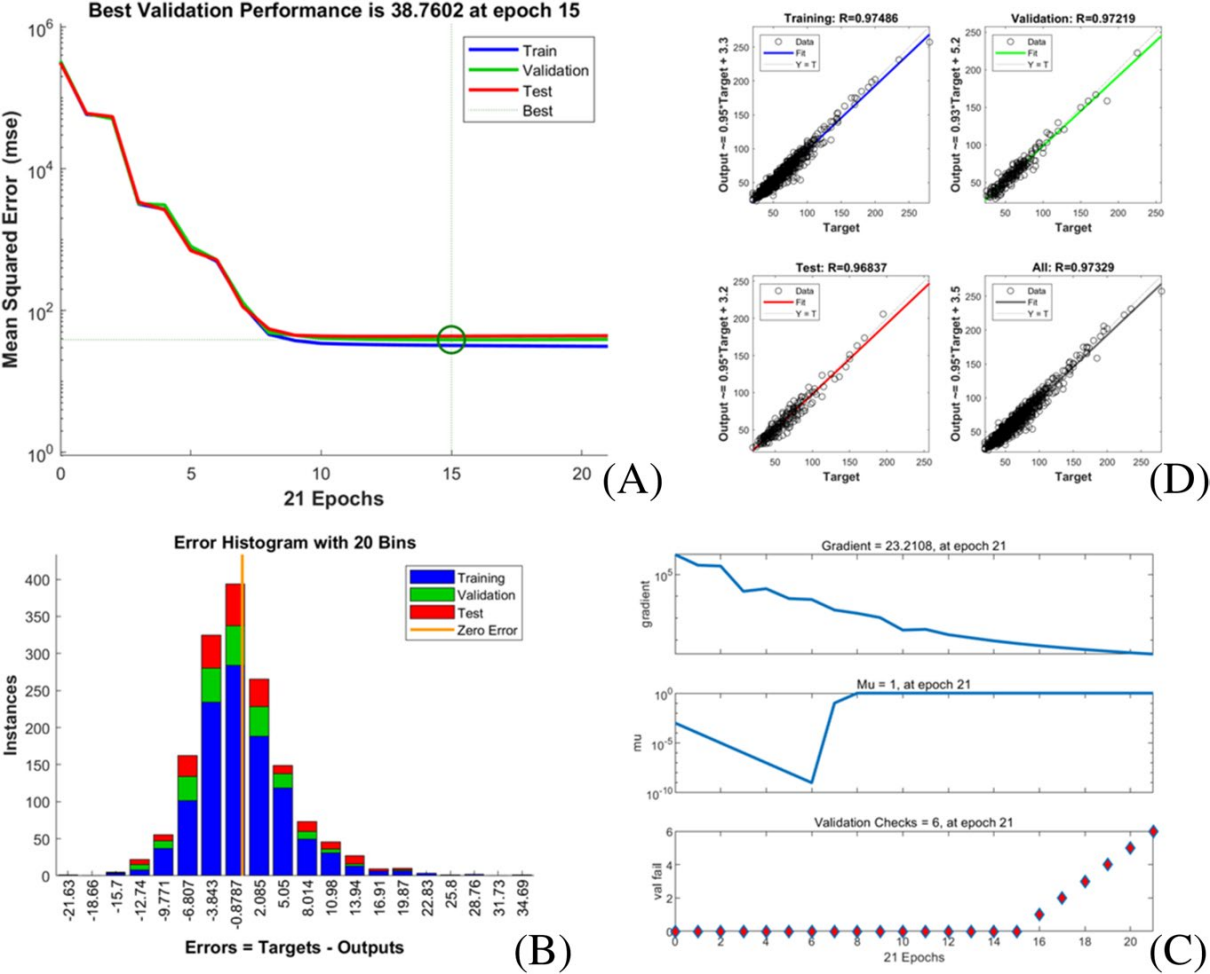
Training state of LM algorithm artificial neural network model of extubation time. **A** performance in LM algorithm; **B** error histogram in LM algorithm; **C** training state in LM algorithm; **D** regression in LM algorithm

**Fig. 4:**
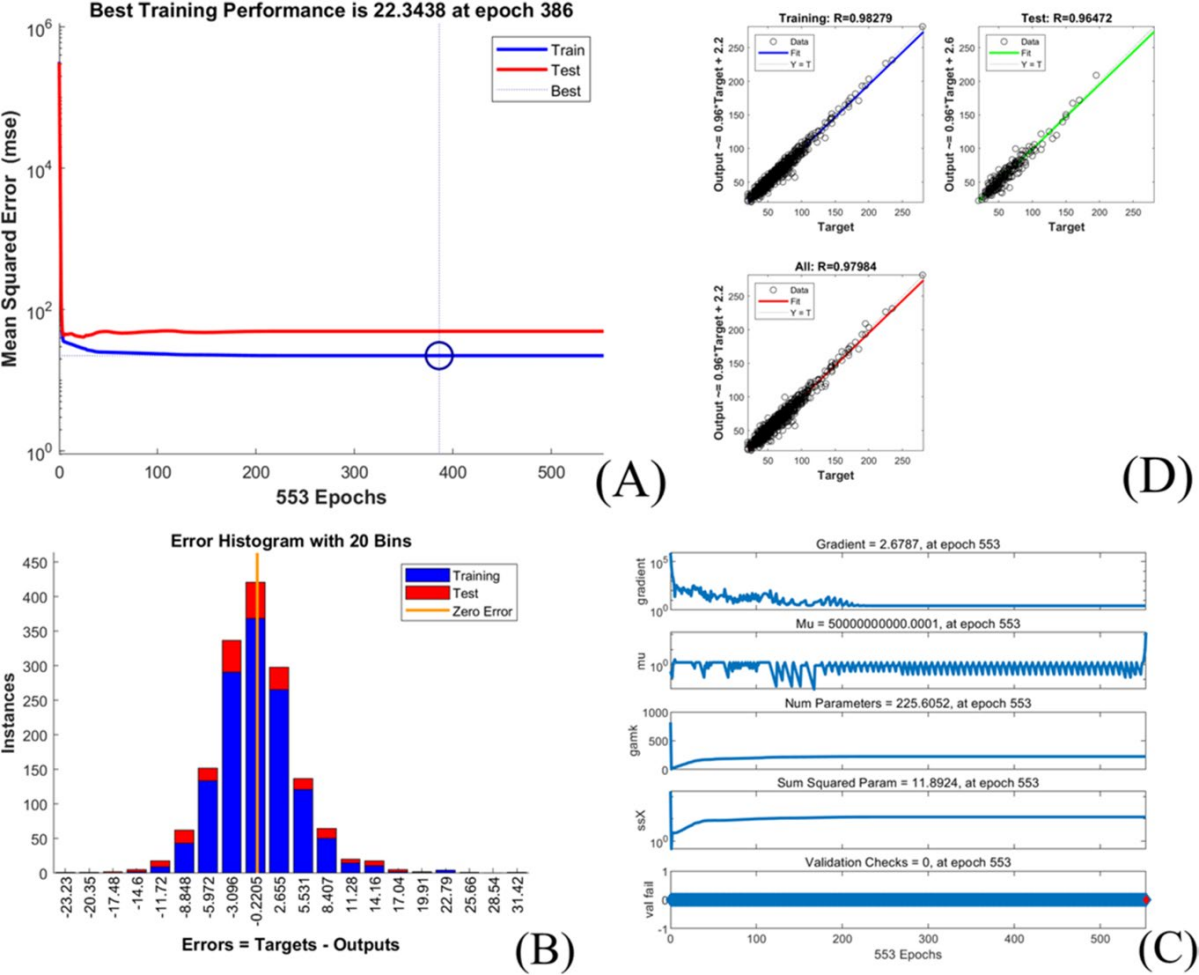
Training state of BR algorithm artificial neural network model of extubation time. **A** performance in BR algorithm; **B** error histogram in BR algorithm; **C** training state in BR algorithm; **D** regression in BR algorithm

**Fig. 5:**
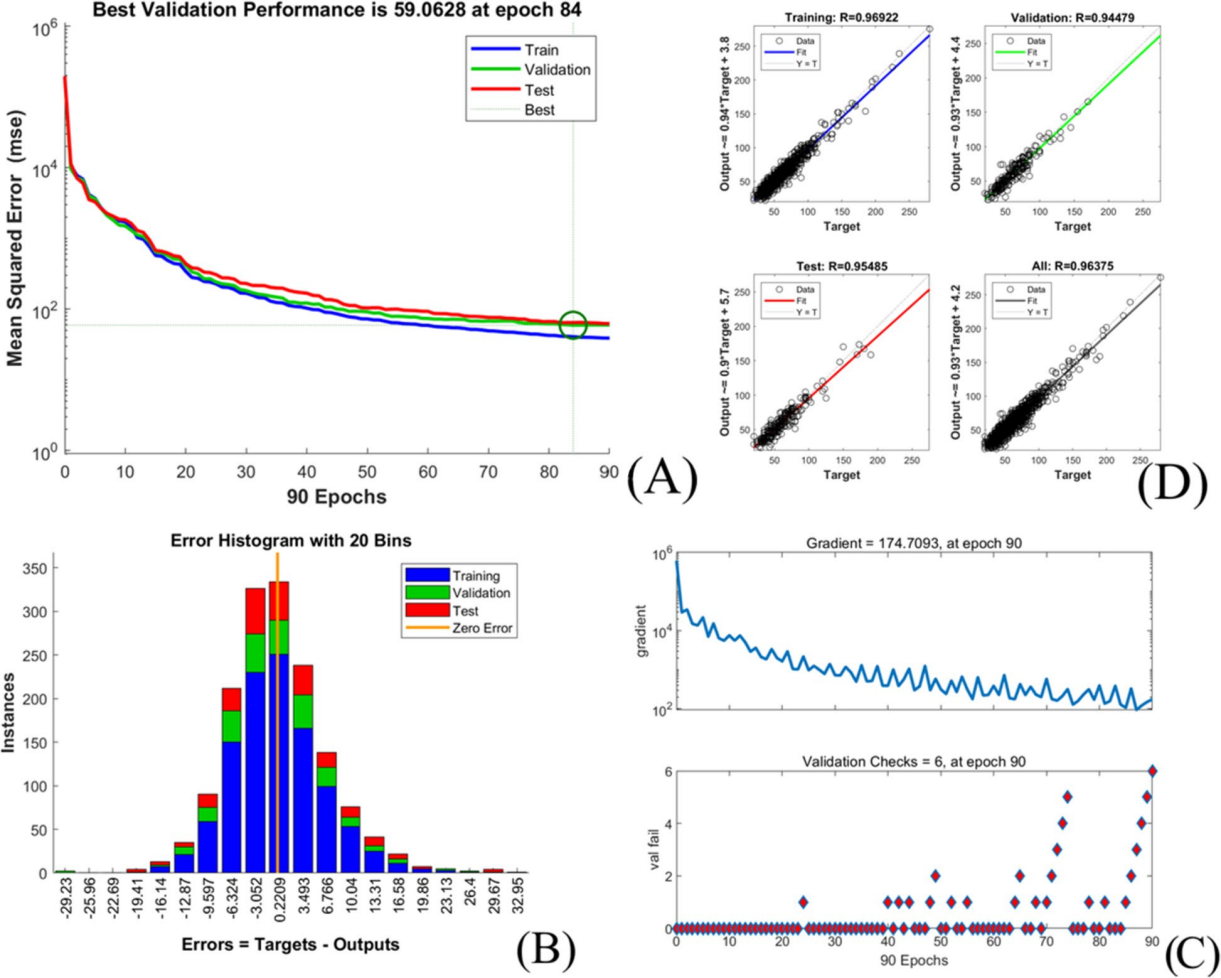
Training state of SCG algorithm artificial neural network model of extubation time. **A** performance in SCG algorithm; **B** error histogram in SCG algorithm; **C** training state in SCG algorithm; **D** regression in SCG algorithm

The extubation time prediction models training performance is showed in Table 4 and Figs. 1, 2, 3, 4, 5.

**Table 4:** Training performance evaluation of extubation time prediction models

Evaluation indexes	FNN	Stepwise linear regression	Regression tree	Ensembles of trees regression	Artificial neural network		
					LM Algorithm	BR Algorithm	SCG Algorithm
MSE	35.055	37.720	61.851	57.566	32.200	22.343	40.798
RMSE	5.921	6.142	7.865	7.587	5.675	4.727	6.387
R-Squared	0.946	0.940	0.910	0.910	0.973	0.980	0.964
Model training time (s)	1.38	669.98	1.78	12.48	1.00	75.00	1.00
Neurons number in the hidden layer	142	-	-	-	39	39	39
Internal verification method	Independent validation dataset	Tenfold cross validation	Tenfold cross validation	Tenfold cross validation	Independent validation dataset	Independent validation dataset	Independent validation dataset

The models’ goodness of fit from high to low are as followed:BRA, LMA, SCGA, FNN, stepwise linear regression model, ensembles of trees regression model and regression tree model with its R-squared value ranged from 0.910 to 0.980, and all models achieved high fitting degree. The models’ accuracy from high to low areBRA, LMA, FNN, stepwise linear regression model, SCGA, ensembles of trees regression model and regression tree model with the RMSE value ranged from 4.727 to 7.865.However, the training time of each model varies greatly, range from 1 s (LMA, SCGA) to 669.98 s (stepwise linear regression model).

#### Generalization performance evaluation

The same check dataset is used to perform generalization test on the trained models respectively, and the generalization results are shown in Table 5.

**Table 5:** Generalization performance evaluation of extubation time prediction model

Evaluation indexes	FNN	Stepwise linear regression	Regression tree	Ensembles of trees regression	Artificial neural network		
					LM Algorithm	BR Algorithm	SCG Algorithm
MSE	44.051	45.943	110.332	101.847	51.040	53.349	62.958
RMSE	6.637	6.778	10.504	10.092	7.144	7.304	7.935
MAE	4.792	4.885	6.308	5.560	5.120	5.419	5.793
MAPE	8.439	8.455	10.151	8.543	8.794	9.337	10.011
R-Squared	0.956	0.954	0.890	0.899	0.949	0.947	0.937
CCC	0.978	0.977	0.945	0.957	0.968	0.963	0.919

All models had exhibited high performancewith the R-squared value between 0.890 ~ 0.956 and the CCC value between 0.919 ~ 0.978. The error between the predicted value and the true value of these models are acceptable with the RMSE value of 6.637 ~ 10.504. Among these models, the fuzzy neural network has the largest R-Squared value and the smallest RMSE value.

### Midterm recovery time prediction model development and evaluation

#### Baseline comparison

The baseline comparison of the training dataset, validation dataset and check dataset for midterm recovery time prediction model is shown in Table 6. Among the 25 predictors included in this study, except for the red blood cell distribution width, the other 24 predictors had no statistical significance among the training dataset, validation dataset and check dataset (*P* > 0.05).

**Table 6:** Baseline comparison of the training dataset, validation dataset and check dataset for midterm recovery time prediction model

Variables	Training dataset (*n* = 1276)	Validation dataset (*n* = 274)	Check dataset (*n* = 274)	Statistic	Sig.
Extubation time (min)	55.00(45.00, 70.00)	60.00(45.00, 75.00)	59.00(45.00, 73.50)	0.232	0.793
Postoperative body temperature (℃)	36.67 ± 0.35	36.65 ± 0.30	36.67 ± 0.32	0.659	0.518
Dexamethasone (mg)	3.00(1.50, 5.00)	2.75(1.50, 5.00)	3.00(2.00, 5.00)	0.261	0.77
Operation time (min)	30.00(20.00, 41.75)	30.00(20.00, 45.00)	30.00(20.00, 42.25)	0.152	0.859
Preoperative atropine (mg)	0.32(0.21, 0.50)	0.35(0.22,0.50)	0.34(0.22,0.50)	0.114	0.892
Nalbuphine (mg)	3.56 ± 1.88	3.55 ± 1.92	3.57 ± 1.90	0.011	0.989
Preoperative body temperature (℃)	36.52 ± 0.22	36.52 ± 0.21	36.51 ± 0.21	0.461	0.63
Transfusion volume (ml)	170.00(121.75, 230.00)	162.50(120.00, 232.50)	170.00(130.00, 220.00)	0.037	0.964
Red blood cell distribution width (%)	11.40(10.90, 12.10)	11.60(11.10, 12.30)	11.30(10.80, 11.90)	4.898	0.008
Postoperative complications (no/yes)	1246/30(97.60%/2.40%)	265/9(96.70%/3.30%)	262/12(95.60%/4.40%)	3.696^a^	0.158
Total carbon dioxide (mmol/L)	22.56 ± 2.46	22.45 ± 2.26	22.65 ± 2.50	0.442	0.643
Underlying diseases (no/yes)	1223/53(95.80%/4.20%)	262/12(95.60%/4.40%)	267/7(97.40%/2.60%)	1.679^a^	0.432
Dexmedetomidine usage (no/yes)	1100/176(86.20%/13.80%)	232/42(84.70%/15.30%)	228/46(83.20%/16.80%)	1.824^a^	0.402
Ondansetron usage (no/yes)	473/803(37.10%/62.90%)	107/167(39.10%/60.90%)	113/161(41.20%/58.80%)	1.819^a^	0.403
ETCO2 (mmHg)	38.17 ± 2.46	38.26 ± 2.66	38.33 ± 2.60	0.509	0.601
Serum total cholesterol (mmol/L)	4.16(3.72, 4.68)	4.23(3.72, 4.76)	4.20(3.75, 4.71)	0.885	0.413
Serum calcium (mmol/L)	2.47 ± 0.16	2.47 ± 0.14	2.46 ± 0.16	0.18	0.835
Muscle relaxant types (atracurium/cisatracurium)	784/492(61.40%/38.60%)	175/99(63.90%/36.10%)	169/105(61.70%/38.30%)	0.566^a^	0.753
Anesthesiologists	-	-	-	0.215^a^	0.898
Surgery types	-	-	-	1.819^a^	0.403
Surgeons	-	-	-	4.323^a^	0.115
Midterm recovery time (min)	72.00(60.00, 90.00)	75.00(60.00, 90.00)	75.00(58.00, 90.00)	0.222	0.801

The statistic is the analysis of variance F value, and ^a^stands for the Chi-square test value

#### Training performance evaluation

**Fig. 6:**
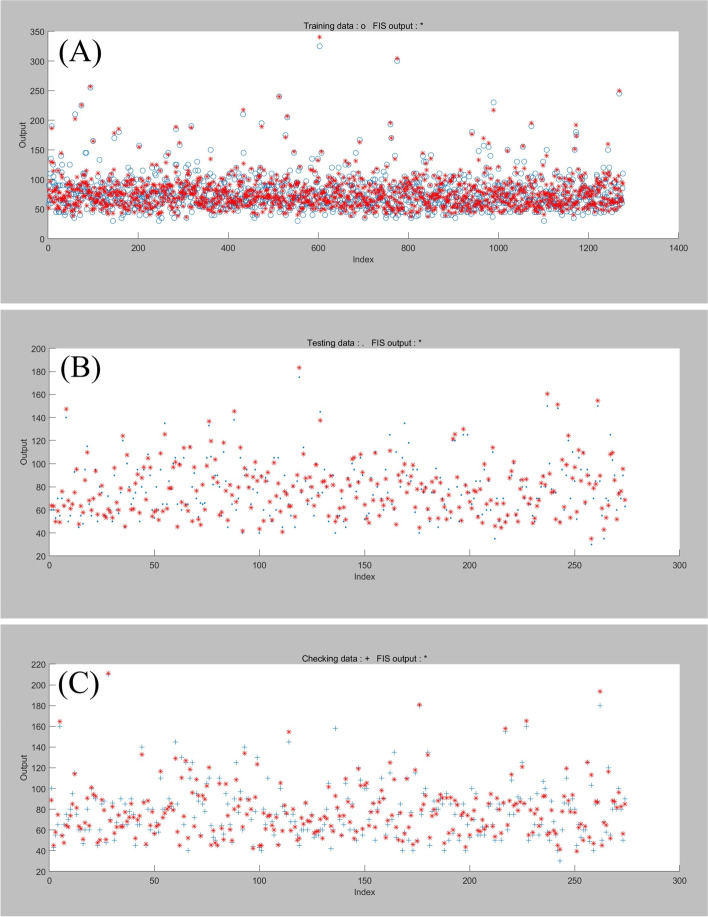
Scatter fitting diagram of predicted and true values of each dataset in midterm recovery time FNN. **A** fitting of the predicted and true value in training dataset; **B** fitting of the predicted and true value in validation dataset; **C** fitting of the predicted and true value in check dataset

**Fig. 7:**
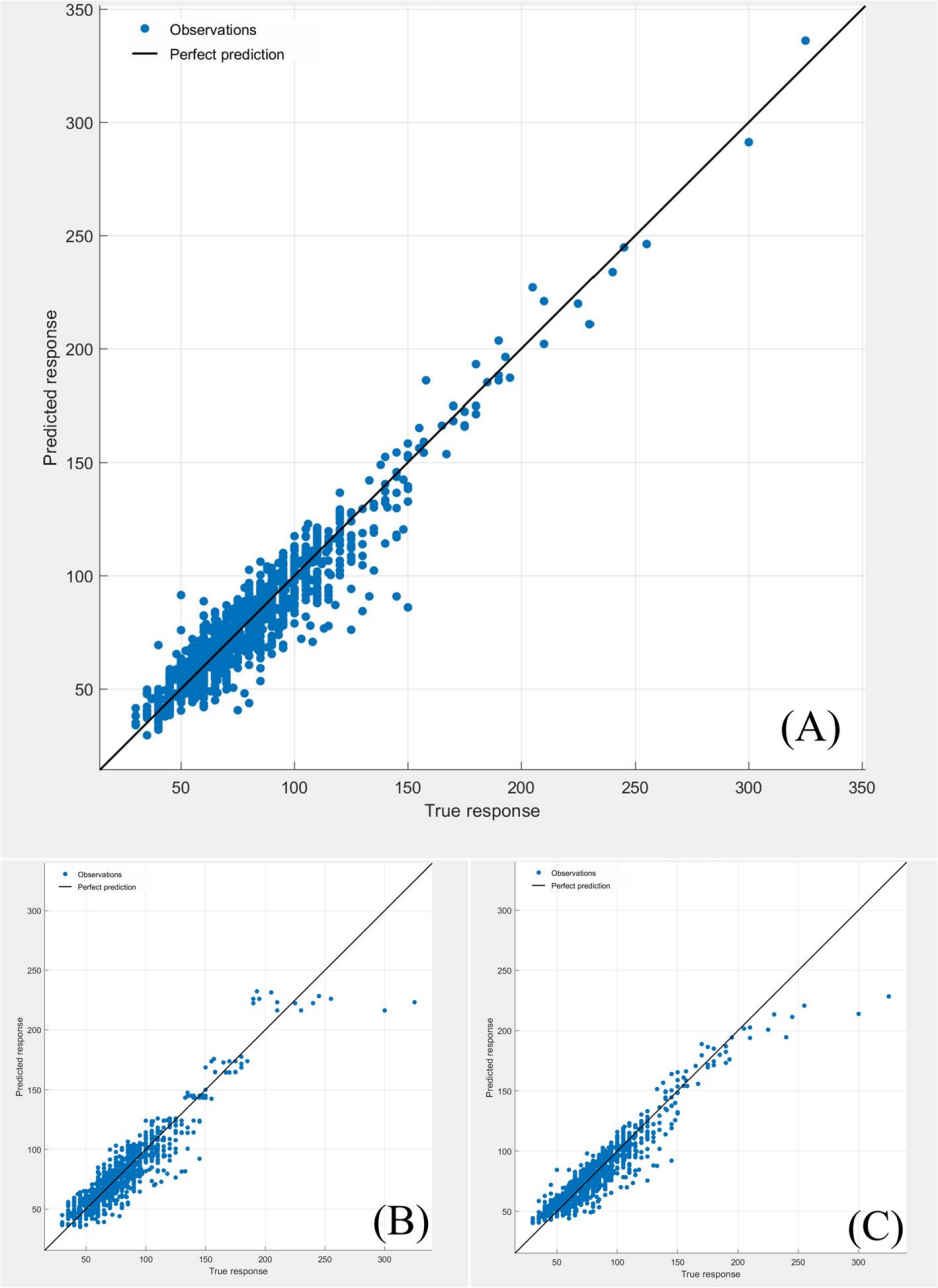
Scatter fitting diagram of predicted and true values in each regression model of midterm recovery time. **A** fitting of the predicted and true value in stepwise linear regression model; **B** fitting of the predicted and true value in regression tree model; **C** fitting of the predicted and true value in ensembles of trees regression model

**Fig. 8:**
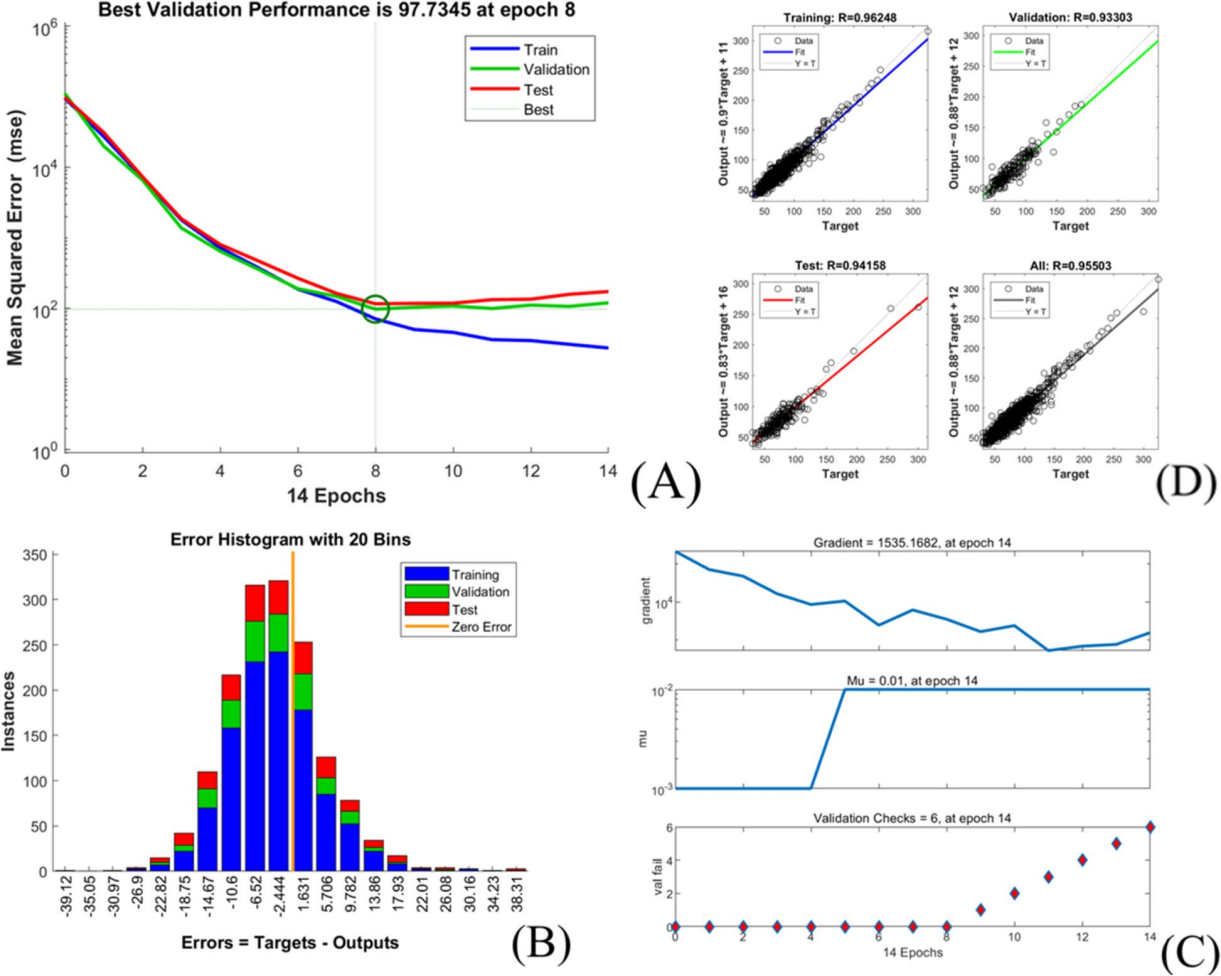
Training state of LM algorithm artificial neural network model of midterm recovery time. **A** performance in LM algorithm; **B** error histogram in LM algorithm; **C** training state in LM algorithm; **D** regression in LM algorithm

**Fig. 9:**
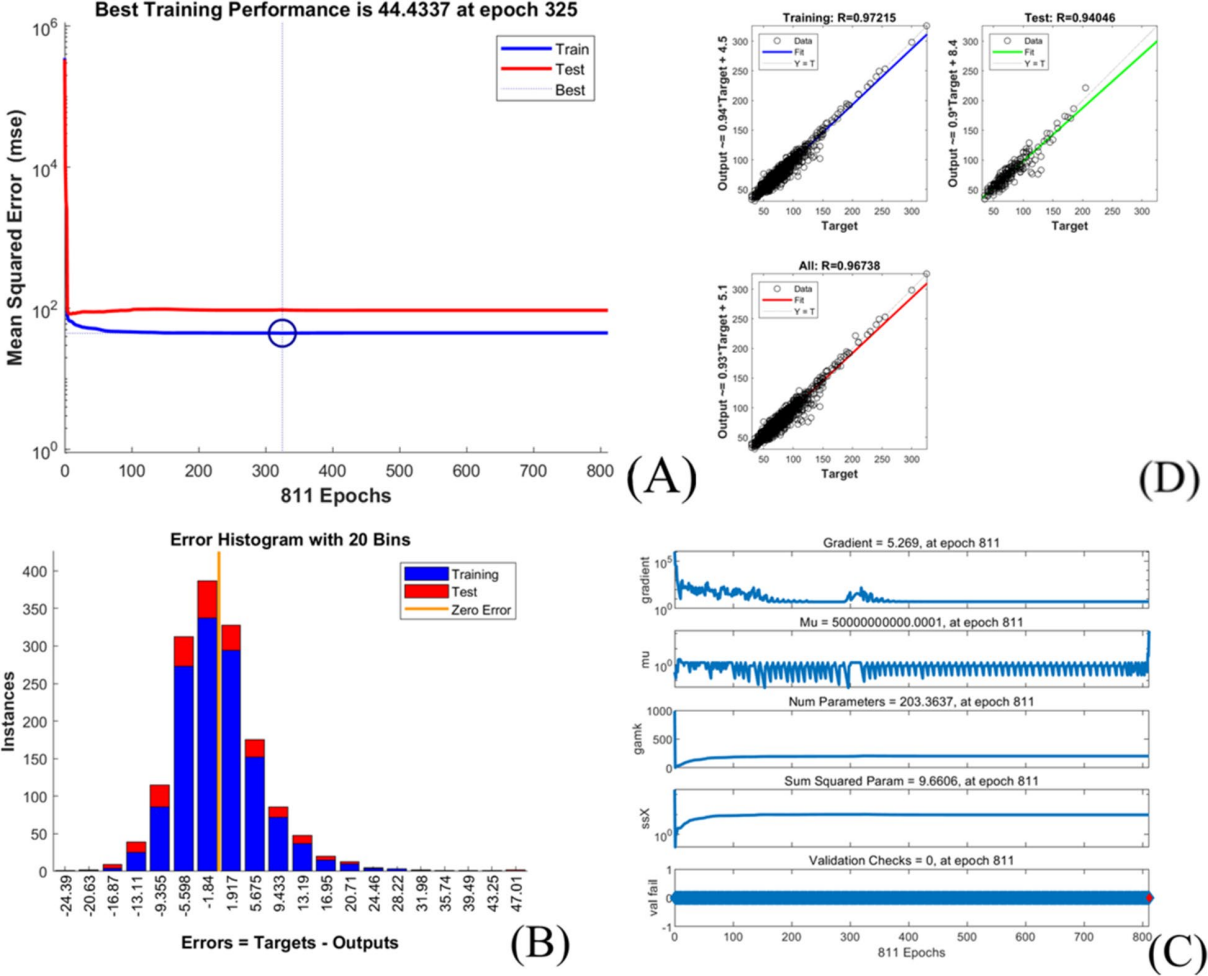
Training state of BR algorithm artificial neural network model of midterm recovery time. **A** performance in BR algorithm; **B** error histogram in BR algorithm; **C** training state in BR algorithm; **D** regression in BR algorithm

**Fig. 10:**
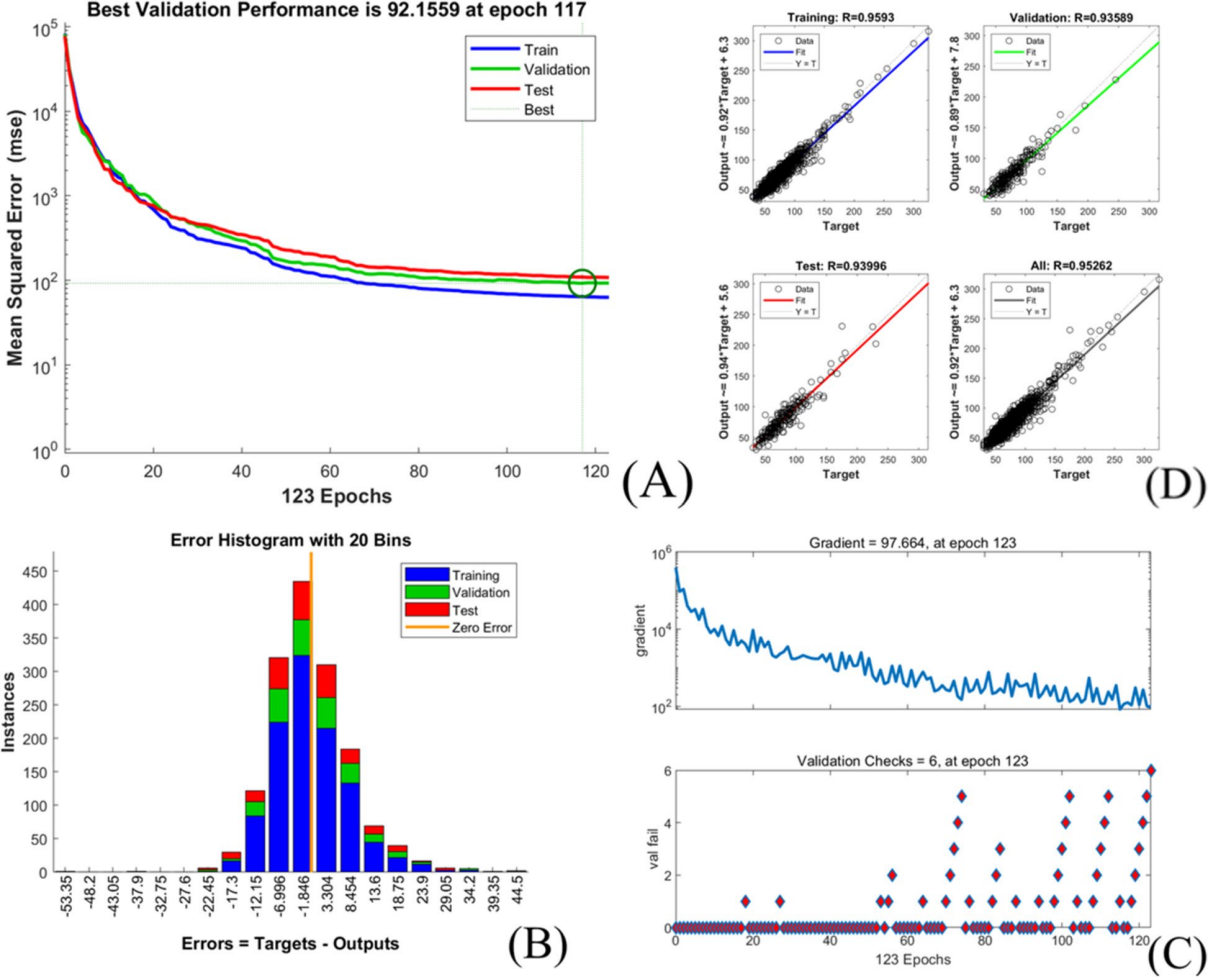
Training state of SCG algorithm artificial neural network model of midterm recovery time. **A** performance in SCG algorithm; **B** error histogram in SCG algorithm; **C** training state in SCG algorithm; **D** regression in SCG algorithm

The midterm recovery time prediction models training performance is showed in Table 7 and Figs. 6, 7, 8, 9, 10.

**Table 7:** Training performance evaluation of midterm recovery time prediction models

Evaluation indexes	FNN	Stepwise linear regression	Regression tree	Ensembles of trees regression	Artificial neural network		
					LM Algorithm	BR Algorithm	SCG Algorithm
MSE	66.265	70.441	105.570	94.873	71.154	44.434	64.081
RMSE	8.140	8.393	10.275	9.740	8.435	6.666	8.005
R-Squared	0.923	0.910	0.870	0.880	0.955	0.967	0.953
Model training time (s)	1.57	956.42	7.56	27.73	1.20	108.00	1.00
neurons number in the hidden layer	112	-	-	-	43	43	43
Internal verification method	Independent validation dataset	tenfold cross validation	tenfold cross validation	tenfold cross validation	Independent validation dataset	Independent validation dataset	Independent validation dataset

The midterm recovery time prediction models’ goodness of fit from high to low are as followed: BRA, LMA, SCGA, FNN, stepwise linear regression model, ensembles of trees regression model and regression tree model with its R-squared value ranged from 0.870 to 0.967, and all models achieved high fitting degree. The models’ accuracy from high to low are BRA, SCGA, FNN, stepwise linear regression model, LMA, ensembles of trees regression model and regression tree model with the RMSE value ranged from 6.666 to 10.275. However, the training time of each model varies greatly, range from 1 s (SCGA) to 956.42 s (stepwise linear regression model).

#### Generalization performance evaluation

The same check dataset is used to perform generalization test on the trained models respectively, and the generalization results are shown in Table 8.

**Table 8:** Generalization performance evaluation of midterm recovery time prediction models

Evaluation indexes	FNN	Stepwise linear regression	Regression tree	Ensembles of trees regression	Artificial neural network		
					LM Algorithm	BR Algorithm	SCG Algorithm
MSE	86.208	95.679	103.158	95.414	142.784	100.649	113.338
RMSE	9.285	9.782	10.157	9.768	11.949	10.032	10.646
MAE	6.557	6.815	6.726	6.608	9.077	7.149	7.653
MAPE	8.849	9.199	8.742	8.791	13.096	9.806	10.294
R-Squared	0.885	0.873	0.863	0.873	0.810	0.866	0.849
CCC	0.941	0.934	0.929	0.934	0.932	0.936	0.921

All models had showed high performancewith the *R*-squared value between 0.810 ~ 0.885 and the CCC value between 0.921 ~ 0.941. The error between the predicted value and the true value of these models are acceptable with the RMSE value of 9.285 ~ 11.949. Among these models, the fuzzy neural network has the largest *R*-Squared value and the smallest RMSE value.

## Discussion

In this study, through comprehensive evaluation of the training performance and generalization results of each model, the FNN gives a more accurate predict results and high calculate performance compared with the other models in predicting extubation time and midterm recovery time of patients underwent general anesthesia. FNN has the characteristics of fast operation speed, high prediction accuracy and strong robustness. Although other models we developed also show good performance in terms of prediction performance and computation speed, only the fuzzy neural network achieves both the high precision prediction efficiency and the fast computation ability at the same time.

Regression model is one of the classical model algorithms, widely used in medicine, finance, industry and other fields [22]. In this study, the prediction performance of stepwise linear regression model is ranked only second to the fuzzy neural network. And three different algorithm artificial neural networks had also showed an acceptable calculation performance.

Among all the extubation time prediction models, the FNN has the smallest absolute value of RMSE in generalization test. The goodness of fit of stepwise linear regression model and FNN are improved in the generalization test, while the BRA fell the most. SCGA has showed the fastest training speed in this study due to its more memory-saving gradient algorithm and fast initial convergence characteristic [23], but it may also lead to large deviations in the output value [24] and reduce its prediction accuracy, robustness and goodness of fit compared with fuzzy neural network.

Among all the midterm recovery time prediction models, the FNN has the smallest absolute value of RMSE in both the training performance and generalization test. And all models’ goodness of fit had decreased compared with the training performance with the LMA has the largest degree of decline. LM algorithm can achieve fast convergence speed and smaller mean square error compared with other algorithms [25], but in terms of practicability, LM algorithm with the increase of neural network weight parameters, the amount of computation required will be geometrically increased, requiring more operational memory and storage space, while the performance of the algorithm decreases [26, 27]. The characteristic of LMA may limit its practical application for it will occupy more operational memory and storage space.

In general, we recommend clinical staff with coding ability use FNN to predict general anesthesia patient’s extubation time or midterm recovery time, and we also considered the regression model and artificial neural network are more suitable for staffs with no coding experience because the Matlab has a built-in tool box for them.

In conclusion, the extubation time prediction models and midterm recovery time prediction models developed in this study have all good predictive abilities (extubation time prediction model R-Squared:0.890–0.956; midterm recovery time prediction model R-Squared:0.810–0.885). In this study, the fuzzy neural network combined with fuzzy theory and artificial neural network can effectively predict both the extubation time and the midterm recovery time of ophthalmic patients underwent general anesthesia with satisfied training speed. Meanwhile, the effectiveness of the fuzzy neural network model is proved by comparing with the classical regression model and the artificial neural network model.

## Limitation

Firstly, the data used in this study were all from one university affiliated tertiary ophthalmic hospital in China and the ASA grade of the patients was in 1 or 2. Therefore, the results of this study have certain limitations and may not be applicable to patients whose ASA grade > 2 or non-ophthalmic patients. Secondly, in the predictors analysis, although we included as many factors as possible by reviewing literature and consulting clinical anesthesiologists, it may not be comprehensive due to the limitations of limited choice in anesthetic agents, disease types and available laboratory test results. Last but not least, the prediction models of extubation time and midterm recovery time for general anesthesia patients constructed in this study has not been externally verified in non-ophthalmic general anesthesia patients, and the applicability of the model still needs further verification. Therefore, we expected to carry out multi-center, multi-diseases and large-sample prospective studies in the future, so as to carry out external validation, strengthen the models’ applicability, and promote its clinical application.

## Implications

The mechanism of delayed emergence from anesthesia is still not fully determined, and there is no specified clinical assessment tool for it. The extubation time and midterm recovery time are the key timings to the early recovery and medium-term recovery periods. In this study, by entering the necessary predictors into the Matlab using the provided codes, the clinical staffs can receive an estimated extubation time and midterm recovery time, which can help them to have an early and accurately identification on high-risk delayed emergence patients. Early intervention for high-risk delayed awakening patients can shorten the duration time in PACU and reduce perioperative risk. Lastly, by predicting patients’ duration time in PACU, administrative staffs can optimize the operation room personnel management and both the bed turnover rate in the operating room and PACU can be improved.

## Supplementary Information

**Additional file 1: Table S1.** Code of new predicted value in different models. **Table S2.** Statistical description of categorical data. **Table S3.** Statistical description of measurement data (conform to normal distribution). **Table S4.** Statistical description of measurement data (not conform to normal distribution). **Table S5.** Categorical variable assignment table. **Table S6.** Summary of multiple linear regression model of extubation time. **Table S7.** Multivariate linear regression model variance analysis of extubation time. **Table S8.** Multiple linear regression analysis of extubation time. **Table S9.** Dummy variable linear regression analysis of extubation time. **Table S10.** Multivariate linear regression model variance analysis of midterm recovery time. **Table S11.** Multivariate linear regression model variance analysis of midterm recovery time. **Table S12.** Multiple linear regression analysis of midterm recovery time. **Table S13.** Dummy variable linear regression analysis of midterm recovery time.

## Data Availability

The data that support the findings of this study are available from Shantou International Eye Centre of Shantou University and the Chinese University of Hong Kong but restrictions apply to the availability of these data, which were used under license for the current study, and so are not publicly available. Data are however available from the corresponding author and the first author upon reasonable request and with permission of Shantou International Eye Centre of Shantou University and the Chinese University of Hong Kong.

## Abbreviations

DEA: Delayed emergence from anesthesia
MSE: Mean square error
RMSE: Root mean square error
R-Squared: Goodness of fit
MAE: Mean absolute error
MAPE: Mean absolute percentage error
PACU: Post-anesthetic care unit
LMA: Levenberg Marquardt Algorithm
BRA: Bayesian Regularization Algorithm
SCGA: Scaled Conjugate Gradient Algorithm
CK-MB: Creatine kinase isoenzyme MB
MCV: Mean corpuscular volume
ETCO2: End-tidal CO2
AI: Artificial intelligence
FNN: Fuzzy neural network
FIS: Fuzzy inference system
