# Supplementary material for: Development of prediction models to estimate extubation time and midterm recovery time of ophthalmic patients undergoing general anesthesia: a cross-sectional study
Source: BMC Anesthesiol. 2023 Mar 17;23:83. doi: 10.1186/s12871-023-02021-3 (PMC10022177; doi:10.1186/s12871-023-02021-3)
Supplement: Supplementary file 1 — Additional file 1: Table S1. Code of new predicted value in different models. Table S2. Statistical description of categorical data. Table S3. Statistical description of measurement data (conform to normal distribution). Table S4. Statistical description of measurement data (not conform to normal distribution). Table S5. Categorical variable assignment table. Table S6. Summary of multiple linear regression model of extubation time. Table S7. Multivariate linear regression model variance analysis of extubation time. Table S8. Multiple linear regression analysis of extubation time. Table S9. Dummy variable linear regression analysis of extubation time. Table S10. Multivariate linear regression model variance analysis of midterm recovery time. Table S11. Multivariate linear regression model variance analysis of midterm recovery time. Table S12. Multiple linear regression analysis of midterm recovery time. Table S13. Dummy variable linear regression analysis of midterm recovery time. [file 12871_2023_2021_MOESM1_ESM.docx]

**1 Calculation code for new predicted value**

**TableS1 Code of new predicted value in different models**

| Model | Code | Description |
| --- | --- | --- |
| Fuzzy neural network | Fis = readfis (‘sugeno211’);  X = data;  Y’ = evalfis (X, fis); | X: new inputs;  Y’: predicted value (i.e., output);  sugeno211: constructed fuzzy inference system. |
| Regression models | X = data;  Y’=trainModel_linear.predictFcn (X); | X: new inputs;  Y’: predicted value (i.e., output);  trainModel_linear: trained regression model. |
| Artificial neural network | X = data;  Y’ =sim (net_trained, X); | X: new inputs;  Y’: predicted value (i.e., output);  net_trained: trained neural network. |

**2 All variables’ statistical description**

**Table S2 Statistical description of categorical data**

| Variables | | Case number (N) | | Constituent ratio (N) |
| --- | --- | --- | --- | --- |
| Gender (male/female) | | 960/864 | | 52.60%/47.40% |
| Timing of surgery (selective/emergency) | | 1767/57 | | 96.90%/3.10% |
| operation history (no/yes) | | 1617/207 | | 88.70%/11.30% |
| Underlying diseases (no/yes) | | 1752/72 | | 96.10%/3.90% |
| ASA grade (degree1/degree2) | | 1766/58 | | 96.80%/3.20% |
| Muscle relaxant types (atracurium/cisatracurium) | | 1128/696 | | 61.80%/38.20% |
| Dexmedetomidine usage (no/yes) | | 1560/264 | | 85.50%/14.50% |
| Ondansetron usage (no/yes) | | 693/1131 | | 38.00%/62.00% |
| Intraoperative atropine (no/yes) | | 1762/62 | | 96.6%/3.4% |
| Urine routine (normal/abnormal) | | 1334/490 | | 73.10%/26.90% |
| Postoperative complications (no/yes) | | 1773/51 | | 97.20%/2.80% |
| Anesthesiologists | |  | |  |
| Chief physician | | 757 | | 41.50% |
| Associate chief physician | | 837 | | 45.90% |
| Attending physician | | 230 | | 12.60% |
| Surgery types | |  | |  |
| Strabismus surgery | | 1494 | | 81.90% |
| Orbital plastic surgery | | 157 | | 8.60% |
| Intraocular surgery 1 | | 88 | | 4.80% |
| Intraocular surgery 2 | | 19 | | 1.00% |
| Intraocular surgery 3 | | 50 | | 2.70% |
| Corneal surgery | | 16 | | 0.90% |
| Surgeons* | |  | |  |
| LSB | | 582 | | 31.90% |
| YF | | 691 | | 37.90% |
| GWF | | 221 | | 12.10% |
| LZY | | 75 | | 4.10% |
| ZYS | | 39 | | 2.10% |
| CJH | | 43 | | 2.40% |
| CHY | | 88 | | 4.80% |
| CWQ | | 19 | | 1.00% |
| ZMZ | | 39 | | 2.10% |
| ZGH | | 11 | | 0.60% |
| XJH | | 16 | | 0.90% |
| * In the variable of surgeons, LSB, YF, GWF, LZY, ZYS, CJH, CHY, CWQ, ZMZ, ZGH, and XJH are initials. | | | | |
| **Table S3 Statistical description of measurement data (conform to normal distribution)** | | | | |
| Variables | Case number (N) | | Mean±Std. Deviation ($\bar{x}$±s) | |
| Height (cm) | 1824 | | 140.34±21.55 | |
| Body mass index (kg/m^2^) | 1824 | | 17.58±3.84 | |
| Preoperative body temperature (℃) | 1824 | | 36.52±0.22 | |
| End-tidal CO2 (mmHg) | 1824 | | 38.21±2.51 | |
| Postoperative body temperature (℃) | 1824 | | 36.67±0.34 | |
| Serum potassium (mmol/L) | 1824 | | 4.23±0.33 | |
| Serum sodium (mmol/L) | 1824 | | 140.97±1.88 | |
| Serum chlorine (mmol/L | 1824 | | 104.92±2.41 | |
| Serum calcium (mmol/L) | 1824 | | 2.47±0.15 | |
| Serum phosphorus (mmol/L) | 1824 | | 1.55±0.28 | |
| Total carbon dioxide (mmol/L) | 1824 | | 22.56±2.43 | |
| Total protein (g/L) | 1824 | | 75.67±5.20 | |
| Albumin (g/L) | 1824 | | 47.31±2.78 | |
| Globulin (g/L) | 1824 | | 28.36±4.17 | |
| ALB | 1824 | | 1.70±0.27 | |
| L-lactate dehydrogenase (U/L) | 1824 | | 230.18±58.99 | |
| Glucose (mmol/L) | 1824 | | 5.55±0.95 | |
| Fructosamine (mmol/L) | 1824 | | 0.24±0.03 | |
| Total cholesterol (mmol/L) | 1824 | | 4.26±0.79 | |
| Neutrophil percentage (%) | 1824 | | 51.41±12.01 | |
| Monocyte percentage (%) | 1824 | | 7.30±1.98 | |
| Red blood cell count (10^12/L) | 1824 | | 5.07±0.51 | |
| Hemoglobin (g/L) | 1824 | | 138.30±14.20 | |
| HCT (%) | 1824 | | 43.40±4.13 | |
| Mean corpuscular volume (fL) | 1824 | | 85.78±7.11 | |
| Mean corpuscular hemoglobin (pg) | 1824 | | 27.37±2.64 | |
| Mean corpuscular hemoglobin concentration(g/L) | 1824 | | 318.87±19.40 | |
| Red blood cell distribution width (%) | 1824 | | 11.71±1.24 | |
| Platelet count (10^9/L) | 1824 | | 284.21±65.37 | |
| Platelet distribution width (%) | 1824 | | 18.93±1.12 | |

**Table S4 Statistical description of measurement data (not conform to normal distribution)**

| Variables | Case number (N) | Median (P25，P75) |
| --- | --- | --- |
| Age (year) | 1824 | 10.00(7.00, 15.00) |
| Weight (kg) | 1824 | 32.45(21.80, 49.50) |
| Preoperative atropine (mg) | 1824 | 0.32(0.22, 0.50) |
| Transfusion volume (ml) | 1824 | 170.00(130.00, 230.00) |
| Surgery time (min) | 1824 | 30.00(20.00, 45.00) |
| Fentanyl (μg) | 1824 | 60.00(40.00, 100.00) |
| Total amount of propofol (mg) | 1824 | 246.84(172.54, 358.63) |
| Total amount of remifentanil (μg) | 1824 | 346.67(200.00, 587.67) |
| Nalbuphine (mg) | 1824 | 3.00(2.00, 5.00) |
| Dexamethasone (mg) | 1824 | 3.00(1.50, 5.00) |
| Tidal volume (ml) | 1824 | 280.00(180.00, 400.00) |
| Anesthesia time (min) | 1824 | 40.00(30.00, 55.00) |
| Extubation time (min) | 1824 | 55.00(45.00, 72.00) |
| Midterm recovery time (min), | 1824 | 75.00(60.00, 90.00) |
| Total bilirubin (μmol/L) | 1824 | 8.10(6.20, 10.70) |
| Direct bilirubin (μmol/L) | 1824 | 2.60(1.80, 3.60) |
| Indirect bilirubin (μmol/L) | 1824 | 5.50(4.10, 7.30) |
| Alanine aminotransferase (U/L) | 1824 | 13.00 (11.00, 17.00) |
| Aspartate aminotransferase (U/L) | 1824 | 24.00 (19.00, 28.00) |
| Glutamyl transpeptidase (U/L) | 1824 | 13.00 (11.00, 17.00) |
| Alkaline phosphatase (U/L) | 1824 | 193.00(103.25, 245.00) |
| Cholinesterase (U/L) | 1824 | 8919.00(7997.00, 9897.75) |
| α-hydroxybutyrate dehydrogenase (U/L) | 1824 | 157.00 (126.00, 186.00) |
| Creatine kinase (U/L) | 1824 | 97.00 (72.00, 131.00) |
| Creatine isoenzyme MB (U/L) | 1824 | 8.00 (4.00, 19.00) |
| Ureophil (mmol/L) | 1824 | 4.35(3.68, 5.15) |
| Creatinine (μ mol/L) | 1824 | 44.00 (36.00, 55.00) |
| Uric acid (μ mol/L) | 1824 | 332.00 (280.00, 401.00) |
| Cystine protease inhibitor C (mg/L) | 1824 | 0.78(0.63, 0.93) |
| Triglyceride (mmol/L) | 1824 | 0.89(0.63, 1.36) |
| High density cholesterol (mmol/L) | 1824 | 1.58(1.37, 1.84) |
| Low density cholesterol (mmol/L) | 1824 | 2.13(1.78, 2.57) |
| C-reactive protein (mg/dL) | 1824 | 0.32(0.00, 0.75) |
| White blood cell count (10^9/L) | 1824 | 7.10(6.10, 8.40) |
| Lymphocyte percentage (%) | 1824 | 38.00(30.23, 44.90) |
| Eosinophil percentage (%) | 1824 | 2.10(1.20, 3.60) |
| Basophils percentage (%) | 1824 | 1.00(0.70, 1.30) |
| Neutrophil absolute value (10^9/L) | 1824 | 3.50(2.70, 4.60) |
| Lymphocyte absolute value (10^9/L) | 1824 | 2.60(2.10, 3.20) |
| Monocyte absolute value (10^9/L) | 1824 | 0.50(0.40, 0.60) |
| Eosinophil absolute value (10^9/L) | 1824 | 0.14(0.08, 0.26) |
| Basophil absolute value (10^9/L) | 1824 | 0.07(0.05, 0.09) |
| Platelet ratio (%) | 1824 | 0.23(0.21, 0.27) |
| Mean platelet volume (fL) | 1824 | 8.40(7.60, 9.40) |

**Table S5 Categorical variable assignment table**

| Variable | Assignment |
| --- | --- |
| Gender | 1: male; 2: female |
| Timing of surgery | 0: selective; 1: emergency |
| Operation history | 0: no; 1: yes |
| Underlying diseases | 0: no; 1: yes |
| ASAgrade | 1: degree1; 2: degree2 |
| Muscle relaxant types | 1: atracurium; 2: cisatracurium |
| Dexmedetomidine usage | 0: no; 1: yes |
| Ondansetron usage | 0: no; 1: yes |
| Intraoperative atropine | 0: no; 1: yes |
| Urine routine | 0: normal; 1: abnormal |
| Postoperative complications | 0: no; 1: yes |
| Anesthesiologists | 1: chief physician  2: associate chief physician  3: attending physician |
| Surgeons* | 1: LSB;  2: YF;  3: GWF;  4: LZY;  5: ZYS;  6: CJH;  7: CHY;  8: CWQ;  9: ZMZ;  10: ZGH;  11: XJH |
| Surgery types | 1: strabismus surgery  2: orbital plastic surgery  3: intraocular surgery 1  4: intraocular surgery 2  5: intraocular surgery 3  6: corneal surgery |
| * In the variable of surgeons, LSB, YF, GWF, LZY, ZYS, CJH, CHY, CWQ, ZMZ, ZGH, and XJH are initials. | |

**3 Risk factors analysis of extubation time**

Multiple linear regression was used to analyze the continuous variables and dichotomous variables affecting the extubation time of patients undergoing general anesthesia. The obtained multiple linear regression model of extubation time included 16 independent variables (P<0.05), and the model calibration R-Squared was 0.947 (**Table S6, S7**). Those independent variables are anesthesia time, Creatine kinase MB isoenzyme (CK-MB), mean corpuscular volume (MCV), ondansetron usage, mean corpuscular hemoglobin concentration, muscle relaxant types, preoperative atropine, nalbuphine, end-tidal CO2 (ETCO2), cystatin, urinalysis, tidal volume, creatinine, platelet ratio, transfusion volume and operation history (**Table S8**). Multivariate linear regression analysis was conducted after dummy variables were set for categorical variables. The independent variables with statistical significance (P<0.05) were anesthesiologists, surgery types and surgeons (**Table S9**) with the calibration R-Squared of 0.044, 0.233 and 0.401, respectively.

**Table S6 Summary of multiple linear regression model of extubation time**

| R | R-square | Adjusted R-square | Std. Error of the Estimate | Durbin-Watson |
| --- | --- | --- | --- | --- |
| 0.973 | 0.947 | 0.947 | 6.176 | 1.642 |

**Table S7 Multivariate linear regression model variance analysis of extubation time**

| Model | Sum of Squares | *df* | Mean Square | *F* | Sig. |
| --- | --- | --- | --- | --- | --- |
| Regression | 1234011.519 | 16 | 77125.720* | 2021.694* | ＜0.001 |
| Residual | 68935.331* | 1807 | 38.149 |  |  |
| Total | 1302946.850* | 1823 |  |  |  |

**Table S8 Multiple linear regression analysis of extubation time**

| Variables | $\bar{x}$±s/M (P25, P75)/% | Unstandardized B* | Coefficients std. error | Standardized coefficients* | Sig. | VIF value* |
| --- | --- | --- | --- | --- | --- | --- |
| Anesthesia time (min) | 40.00(30.00, 55.00) | 0.984 | 0.007 | 0.938 | ＜0.001 | 1.371 |
| CK-MB (U/L) | 8.00(4.00, 19.00) | 0.174 | 0.018 | 0.061 | ＜0.001 | 1.423 |
| MCV (fL) | 85.78±7.11 | 0.089 | 0.022 | 0.024 | ＜0.001 | 1.176 |
| Ondansetron usage (no/yes) | 38.00%/62.00% | -1.413 | 0.308 | -0.026 | ＜0.001 | 1.068 |
| Mean corpuscular hemoglobin concentration (g/L) | 318.87±19.40 | -0.023 | 0.008 | -0.016 | 0.004 | 1.076 |
| Muscle relaxant types (atracurium/cisatracurium) | 61.80%/38.20% | -1.409 | 0.303 | -0.026 | ＜0.001 | 1.033 |
| Preoperative atropine (mg) | 0.32(0.22, 0.50) | -7.479 | 2.455 | -0.039 | 0.002 | 5.703 |
| Nalbuphine (mg) | 3.00 (2.00, 5.00) | 1.093 | 0.152 | 0.077 | ＜0.001 | 3.922 |
| ETCO2 (mmHg) | 38.21±2.51 | -0.183 | 0.059 | -0.017 | 0.002 | 1.054 |
| Cystatin (mg/L) | 0.78(0.63, 0.93) | 2.783 | 0.858 | 0.021 | 0.001 | 1.439 |
| Urine routine (normal/abnormal) | 73.10%/26.90% | 0.923 | 0.334 | 0.015 | 0.006 | 1.048 |
| Tidal volume (ml) | 280.00 (180.00, 400.00) | -0.014 | 0.004 | -0.064 | ＜0.001 | 8.271 |
| Creatinine (μmol/L) | 44.00 (36.00, 55.00) | 0.048 | 0.015 | 0.028 | 0.002 | 2.725 |
| Platelet ratio (%) | 0.23(0.21, 0.27) | 8.484 | 3.194 | 0.015 | 0.008 | 1.071 |
| Transfusion volume (ml) | 170.00 (130.00, 230.00) | 0.004 | 0.002 | 0.016 | 0.011 | 1.348 |
| Operation history (no/yes) | 88.70%/11.30% | 1.150 | 0.460 | 0.014 | 0.012 | 1.018 |
| * VIF: stands for variance inflation factor. | | | | | | |

**Table S9 Dummy variable linear regression analysis of extubation time**

| Variables | Proportion (%) | Unstandardized B* | Coefficients std. error | Standardized coefficients* | Sig. | VIF value* |
| --- | --- | --- | --- | --- | --- | --- |
| Anesthesiologists |  |  |  |  |  |  |
| Associate chief physician | 45.90% | -17.335 | 1.947 | -0.323 | ＜0.001 | 2.510 |
| Chief physician | 41.50% | -16.568 | 1.969 | -0.305 | ＜0.001 | 2.510 |
| Surgery types |  |  |  |  |  |  |
| Orbital plastic surgery | 8.60% | 40.914 | 1.975 | 0.429 | ＜0.001 | 1.006 |
| Corneal surgery | 0.90% | 52.749 | 5.927 | 0.184 | ＜0.001 | 1.001 |
| Intraocular surgery 1 | 4.80% | 16.828 | 2.584 | 0.135 | ＜0.001 | 1.005 |
| Surgeons** |  |  |  |  |  |  |
| LSB | 31.90% | -47.377 | 2.365 | -0.826 | ＜0.001 | 5.134 |
| YF | 37.90% | -28.194 | 2.340 | -0.512 | ＜0.001 | 5.444 |
| LZY | 4.10% | 12.941 | 3.257 | 0.096 | ＜0.001 | 1.767 |
| ZMZ | 2.10% | -34.780 | 3.990 | -0.188 | ＜0.001 | 1.407 |
| ZYS | 2.10% | 9.426 | 3.990 | 0.051 | 0.018 | 1.407 |
| GWF | 12.10% | -15.065 | 2.608 | -0.184 | ＜0.001 | 3.061 |
| CHY | 4.80% | -16.392 | 3.124 | -0.131 | ＜0.001 | 1.893 |
| * VIF: stands for variance inflation factor.  ** In the variable of surgeons, LSB, YF, LZY, ZMZ, ZYS, GWF, CHY are initials. | | | | | | |

**4 Risk factors analysis of midterm recovery time**

Multiple linear regression was used to analyze the continuous variables and dichotomous variables affecting the midterm recovery time of patients undergoing general anesthesia. The obtained multiple linear regression model of extubation time included 18 independent variables (P<0.05), and the model calibration R-Squared was 0.91 (**Table S10, S11**). Those independent variables are extubation time, postoperative body temperature, dexamethasone, operation time, preoperative atropine, nalbuphine, preoperative body temperature, transfusion volume, red blood cell distribution width, postoperative complications, total carbon dioxide, underlying diseases, dexmedetomidine usage, ondansetron usage, ETCO2, serum total cholesterol, serum calcium and muscle relaxant types (**Table S12**). Multivariate linear regression analysis was conducted after dummy variables were set for categorical variables. The independent variables with statistical significance (P<0.05) were anesthesiologists, surgery types and surgeons (**Table S13**) with the calibration R-Squared of 0.031, 0.243 and 0.401, respectively.

**Table S10 Multivariate linear regression model variance analysis of midterm recovery time**

| R | R-square | Adjusted R-square | Std. Error of the Estimate | Durbin-Watson |
| --- | --- | --- | --- | --- |
| 0.954 | 0.911 | 0.910 | 8.512 | 1.560 |

**Table S11 Multivariate linear regression model variance analysis of midterm recovery time**

| Model | Sum of Squares | *df* | Mean Square | *F* | Sig. |
| --- | --- | --- | --- | --- | --- |
| Regression | 1331102.204 | 18 | 73950.122* | 1020.620* | ＜0.001 |
| Residual | 130783.203 | 1805 | 72.456* |  |  |
| Total | 1461885.407 | 1823 |  |  |  |

**Table S12 Multiple linear regression analysis of midterm recovery time**

| Variables | $\bar{x}$±s/M (P25, P75)/% | Unstandardized B* | Coefficients std. error | Standardized coefficients* | Sig. | VIF value* |
| --- | --- | --- | --- | --- | --- | --- |
| Extubation time (min) | 55.00 (45.00, 72.00) | 0.847 | 0.020 | 0.800 | ＜0.001 | 7.483 |
| Postoperative body temperature (℃) | 36.67±0.34 | 2.901 | 0.639 | 0.035 | ＜0.001 | 1.168 |
| Dexamethasone (mg) | 3.00(1.50, 5.00) | 0.765 | 0.140 | 0.051 | ＜0.001 | 1.771 |
| Operation time (min) | 30.00 (20.00, 45.00) | 0.147 | 0.022 | 0.123 | ＜0.001 | 7.046 |
| Preoperative atropine (mg) | 0.32(0.22, 0.50) | -17.364 | 2.460 | -0.086 | ＜0.001 | 3.016 |
| Nalbuphine (mg) | 3.00 (2.00, 5.00) | 0.780 | 0.196 | 0.052 | ＜0.001 | 3.441 |
| Preoperative body temperature (℃) | 36.52±0.22 | 3.023 | 0.969 | 0.023 | ＜0.001 | 1.113 |
| Transfusion volume (ml) | 170(130, 230) | 0.008 | 0.002 | 0.029 | ＜0.001 | 1.354 |
| Red blood cell distribution width (%) | 11.71±1.24 | -0.497 | 0.168 | -0.022 | ＜0.001 | 1.091 |
| Postoperative complications (no/yes) | 97.20%/2.80% | 4.282 | 1.229 | 0.025 | ＜0.001 | 1.033 |
| Total carbon dioxide (mmol/L) | 22.56±2.43 | 0.356 | 0.088 | 0.031 | ＜0.001 | 1.148 |
| Underlying diseases (no/yes) | 96.10%/3.90% | 3.746 | 1.047 | 0.026 | ＜0.001 | 1.045 |
| Dexmedetomidine usage (no/yes) | 85.50%/14.50% | -3.701 | 0.710 | -0.046 | ＜0.001 | 1.571 |
| Ondansetron usage (no/yes) | 38.00%/62.00% | -2.225 | 0.486 | -0.038 | ＜0.001 | 1.398 |
| ETCO2 (mmHg) | 38.21±2.51 | 0.249 | 0.082 | 0.022 | ＜0.001 | 1.060 |
| Serum total cholesterol (mmol/L) | 4.26±0.79 | -0.780 | 0.256 | -0.022 | ＜0.001 | 1.035 |
| Serum calcium (mmol/L) | 2.47±0.15 | 4.439 | 1.393 | 0.024 | ＜0.001 | 1.152 |
| Muscle relaxant types (atracurium/cisatracurium) | 61.80%/38.20% | 1.211 | 0.425 | 0.021 | ＜0.001 | 1.072 |
| * VIF: stands for variance inflation factor. | | | | | | |

**Table S13 Dummy variable linear regression analysis of midterm recovery time**

| Variables | Proportion (%) | Unstandardized B* | Coefficients std. error | Standardized coefficients* | Sig. | VIF value* |
| --- | --- | --- | --- | --- | --- | --- |
| Anesthesiologists |  |  |  |  |  |  |
| Associate chief physician | 45.90% | -16.108 | 2.075 | -0.284 | ＜0.001 | 2.510 |
| Chief physician | 41.50% | -13.864 | 2.098 | -0.241 | ＜0.001 | 2.510 |
| Surgery types |  |  |  |  |  |  |
| Orbital plastic surgery | 8.60% | 44.200 | 2.065 | 0.438 | ＜0.001 | 1.007 |
| Corneal surgery | 0.90% | 57.233 | 6.195 | 0.189 | ＜0.001 | 1.001 |
| Intraocular surgery 1 | 4.80% | 19.631 | 2.702 | 0.149 | ＜0.001 | 1.006 |
| Intraocular surgery 2 | 1.00% | 32.720 | 5.691 | 0.117 | ＜0.001 | 1.002 |
| Surgeons** |  |  |  |  |  |  |
| LSB | 31.90% | -50.803 | 2.505 | -0.836 | ＜0.001 | 5.134 |
| YF | 37.90% | -30.558 | 2.479 | -0.524 | ＜0.001 | 5.444 |
| LZY | 4.10% | 13.787 | 3.450 | 0.097 | ＜0.001 | 1.767 |
| ZYS | 2.10% | 8.794 | 4.227 | 0.045 | 0.038 | 1.407 |
| ZMZ | 2.10% | -34.488 | 4.227 | -0.176 | ＜0.001 | 1.407 |
| GWF | 12.10% | -17.934 | 2.763 | -0.207 | ＜0.001 | 3.061 |
| CHY | 4.80% | -16.712 | 3.309 | -0.126 | ＜0.001 | 1.893 |
| * VIF: stands for variance inflation factor.  ** In the variable of surgeons, LSB, YF, LZY, ZMZ, ZYS, GWF, CHY are initials. | | | | | | |
